# Determination of Enantiomeric Excess by Solid-Phase Extraction Using a Chiral Metal-Organic Framework as Sorbent

**DOI:** 10.3390/molecules23112802

**Published:** 2018-10-29

**Authors:** Jun-Hui Zhang, Bo Tang, Sheng-Ming Xie, Bang-Jin Wang, Mei Zhang, Xing-Lian Chen, Min Zi, Li-Ming Yuan

**Affiliations:** 1 Department of Chemistry, Yunnan Normal University, Kunming 650500, China; zjh19861202@126.com (J.-H.Z.); tangbobengbu@163.com (B.T.); xieshengming_2006@163.com (S.-M.X.); wangbangjin711@163.com (B.-J.W.); zimin48@126.com (M.Z.); 2 College of Pharmaceutical Science, Yunnan University of Traditional Chinese Medicine, Kunming 650500, China; meizhang213@163.com; 3 Institute of Quality Standards and Testing Technology, Yunnan Academy of Agricultural Sciences, Kunming 650224, China; chenxinglian@126.com

**Keywords:** metal-organic frameworks, enantiomeric excess, chiral compounds, solid-phase extraction

## Abstract

Metal-organic frameworks (MOFs) have recently attracted considerable attention because of their fascinating structures and intriguing potential applications in diverse areas. In this study, we developed a novel method for determination of enantiomeric excess (*ee*) of (±)-1,1′-bi-2-naphthol by solid-phase extraction (SPE) using a chiral MOF, [Co(l-tyr)]*_n_*(l-tyrCo), as sorbent. After optimization of the experimental conditions, a good linear relationship between the *ee* and the absorbance of the eluate (*R*^2^ = 0.9984) was obtained and the standard curve was established at the concentration of 3 mmol L^−^^1^. The *ee* values of (±)-1,1′-bi-2-naphthol samples can be rapidly calculated using the standard curve after determination of the absorbance of the eluate. The method showed good accuracy, with an average error of 2.26%, and is promising for *ee* analysis.

## 1. Introduction

Optical purity is important in many industries, particularly in the development and production of pharmaceuticals. In general, one enantiomer of chiral drugs is safe and effective, while the other is may be ineffective or even toxic [1]. Consequently, the determination of enantiomeric excess (*ee*) of chiral compounds is very important. At present, chiral chromatographic methods, such as high performance liquid chromatography (HPLC) and gas chromatography (GC) using chiral stationary phases (CSPs), are most commonly used to determine *ee* values. However, these methods have some drawbacks, such as the high cost of chiral columns and long analysis times. Therefore, the development of simple and fast methods for *ee* analysis has great value. A number of techniques for rapid *ee* analysis have been developed, including circular dichroism [2,3], UV and colorimetric methods [4,5], fluorescence spectroscopy [6,7], mass spectrometry [8], and molecularly imprinted polymer based assays [9].

Solid-phase extraction (SPE) is a valuable sample pretreatment technique that enables concentration and purification of analytes from complex matrices. It has been widely used for its simplicity, rapidity, low cost, and compatibility with different detection techniques in both on-line and off-line modes [10,11]. The adsorbent is the core in SPE. High enrichment efficiency and selectivity of SPE are particularly dependent on the adsorbent. Thus, development of novel and efficient adsorbents for SPE is an ongoing research interest.

Metal-organic frameworks (MOFs) are an emerging class of crystalline porous materials that combine metal nodes with organic linkers through coordination bonds [12,13]. MOFs have attracted considerable attention in recent years because of their fascinating structures and outstanding properties, such as permanent nanoscale porosity, high surface area, good chemical stability, rich topology, and tunable pore size. These unique features make MOFs attractive as advanced media for separation [14,15], gas adsorption and storage [16,17], catalysis [18,19], sensing [20,21], imaging [22], and other applications. To date, a number of MOFs, such as MIL-53 [23,24], MIL-100 [23], MIL-101 [23,25,26], MOF-199 [27,28], ZIF-8 [29,30], ZIF-67 [31], ZIF-90 [32], MIL-88B [33], MAF-X8 [34], MOF-5 [35], UIO-66 [36], and UIO-67 [37], have been used as adsorbents for SPE or solid-phase microextraction (SPME) and shown much promise [14,38,39].

**Figure 1:**
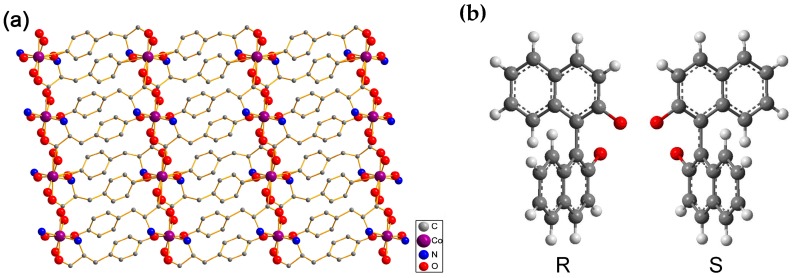
(**a**) 2D crystal structure of the chiral metal-organic framework (MOF), [Co(l-tyr)]*_n_*(l-tyrCo); (**b**) the structures of 1,1′-bi-2-naphthol enantiomers.

The chiral 3D MOF, [Co(l-tyr)]*_n_*(l-tyrCo), composed of metal dimers (Co^II^) linked via l-tyrosine ligand (Figure 1a) [40]. In this paper, we report the determination of *ee* values of (±)-1,1′-bi-2-naphthol (Figure 1b) by SPE using [Co(l-tyr)]*_n_*(l-tyrCo) as the adsorbent. The experimental results showed that the *ee* values of (±)-1,1′-bi-2-naphthol were linearly correlated with the absorbance of the eluates, and a standard curve could be established for *ee* analysis. The *ee* values of (±)-1,1′-bi-2-naphthol samples can be rapidly measured using the standard curve by determining the absorbance of the eluates after SPE. The method has advantages that include of good accuracy, low cost, simple operation, and suitability for batch analysis.

## 2. Results and Discussion

### 2.1. Characterization of the Synthesized [Co(l-tyr)]_n_(l-tyrCo)

**Figure 2:**
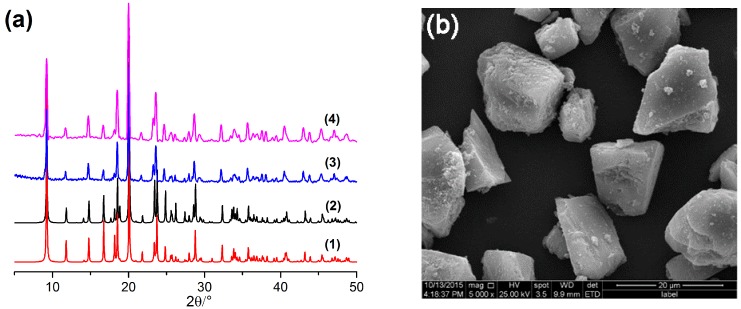
(**a**) Powder X-ray diffraction (PXRD) patterns of [Co(l-tyr)]*_n_*(l-tyrCo): (1) simulated from single crystal X-ray diffraction data, (2) as-synthesized, (3) after the experiment, (4) after soaking in a large volume of methanol and n-hexane/isopropanol (70/30, *v*/*v*) for 48 h; (**b**) SEM image of the selected [Co(l-tyr)]*_n_*(l-tyrCo) particles.

**Figure 3:**
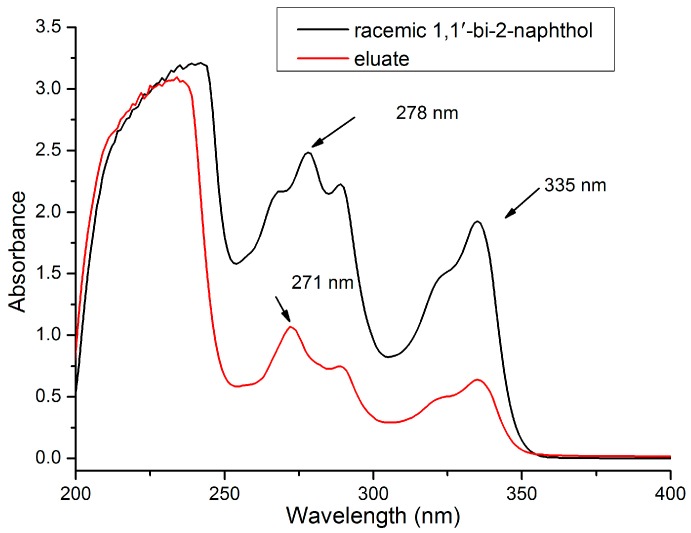
UV-visible spectra of racemic (±)-1,1′-bi-2-naphthol and eluate.

**Figure 4:**
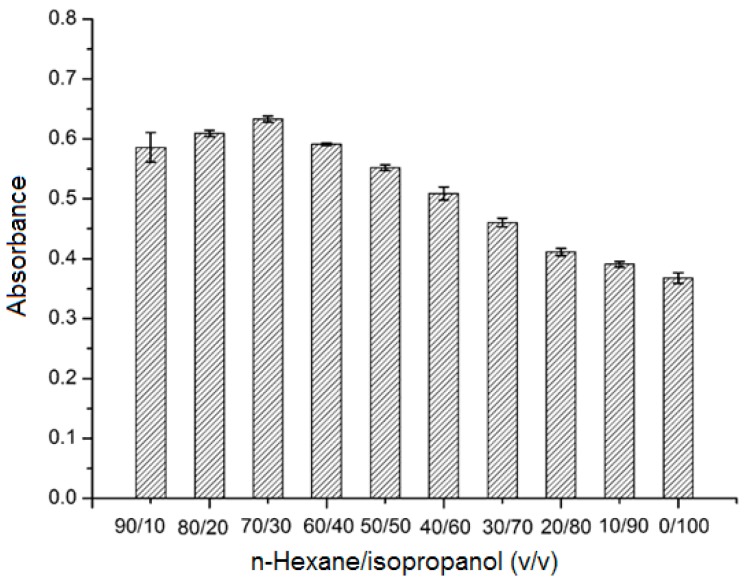
Effect of solvent composition. Analyte concentration: 3.0 mmol L^−1^; sample volume: 2.0 mL; and elution solvent: methanol (4 mL).

The synthesized [Co(l-tyr)]*_n_*(l-tyrCo) was characterized by powder X-ray diffraction (PXRD). As shown in Figure 2a, the experimental PXRD pattern of the synthesized [Co(l-tyr)]*_n_*(l-tyrCo) crystals was in good agreement with that simulated from single crystal data, indicating successful synthesis. After several extraction experiments and soaking in a large volume of methanol and *n*-hexane/isopropanol (70/30, *v*/*v*) for 48 h, the PXRD pattern was not significantly changed, suggesting good stability of the MOF, and that it would not be dissolved during the experiments Figure 2a, (3) and (4). The scanning electron microscopy (SEM) image showed the selected crystals of [Co(l-tyr)]*_n_*(l-tyrCo) with an average size of about 10 μm (Figure 2b).

### 2.2. Selection of UV Wavelength for Analysis of Eluate

To obtain the highest sensitivity, the optimum UV absorption wavelength of the analyte should be determined. The UV-visible absorption spectrum of (±)-1,1′-bi-2-naphthol in methanol was measured over 200–400 nm. As can be seen in Figure 3, the solution of (±)-1,1′-bi-2-naphthol showed strong absorptions at 278 and 335 nm. In principle, both of these wavelengths could be used to determine the absorbance of the eluate. However, some impurities in the eluent absorbed at around 271 nm, which would affect the accuracy of the experimental result. In contrast, impurities in the eluent had relatively low absorption at 335 nm, so this wavelength was used in subsequent experiments.

### 2.3. Optimization of SPE Conditions

The sorption of (±)-1,1′-bi-2-naphthol on a SPE cartridge packed with [Co(l-tyr)]*_n_*(l-tyrCo) can be influenced by many experimental conditions, such as solvent composition, sample solution volume, analyte concentration, and the type and volume of eluent. To obtain good extraction efficiency, these parameters were optimized. 

#### 2.3.1. Effect of Sample Solvent Composition

The sample solvent composition will influence adsorption of the analyte on SPE adsorbent. In this study, (±)-1,1′-bi-2-naphthol was dissolved in mixtures of *n*-hexane/isopropanol mixtures at different ratios (90/10, 80/20, 70/30, 60/40, 50/50, 40/60, 30/70, 20/80, 10/90, and 0/100; *v*/*v*). As can be seen in Figure 4, the maximum absorbance of the eluate was obtained when using *n*-hexane/isopropanol = 70/30 (*v*/*v*) as solvent. This solvent mixture was therefore chosen for the following experiments.

#### 2.3.2. Effect of Sample Solution Volume

**Figure 5:**
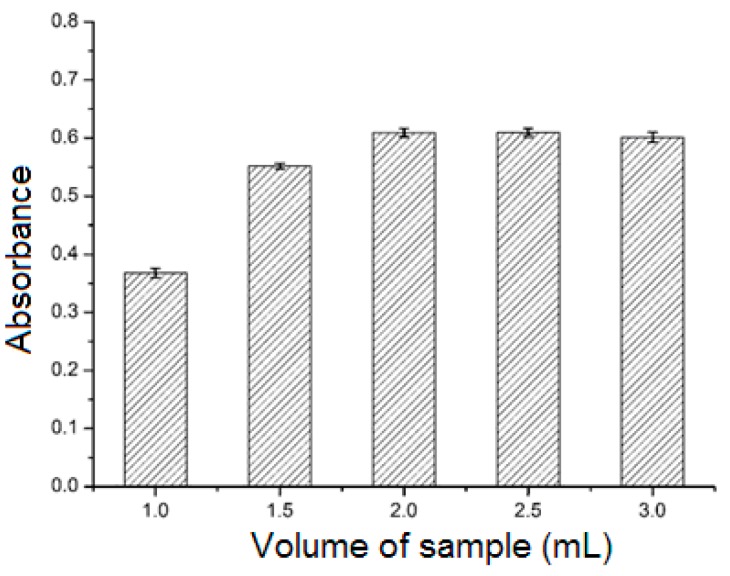
Effect of sample solution volume. Analyte concentration: 3.0 mmol L^−1^; elution solvent: methanol (4 mL).

The adsorption efficiency can also be influenced by the volume of the sample solution. Volumes of sample solution (3 mmol L^−1^) in the range of 1.0–3.0 mL were passed through the SPE cartridge, and the adsorbed analytes were eluted with methanol (4 mL). As shown in Figure 5, the absorbance of the eluate increased as the sample solution volume was increased from 1.0 mL to 2.0 mL, but remained stable as the volume was increased to 3.0 mL. Thus, the optimum volume was 2.0 mL.

#### 2.3.3. Effect of Analyte Concentration 

**Figure 6:**
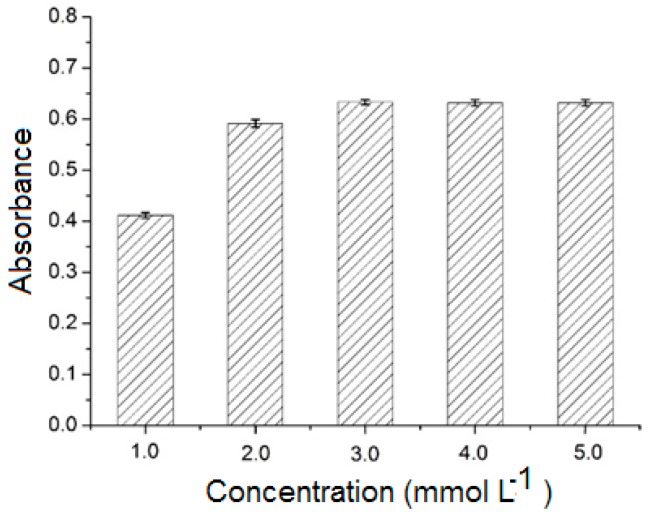
Effect of analyte concentration. Sample volume: 2.0 mL; and elution solvent: methanol (10 mL).

Five different concentrations (1.0, 2.0, 3.0, 4.0, and 5.0 mmol L^−1^) of (±)-1,1′-bi-2-naphthol were investigated. As shown in Figure 6, the absorbance of the eluate increased as the analyte concentration was increased from 1.0 to 3.0 mmol L^−1^, with no further increase as the concentration was raised to 5.0 mmol L^−1^. The optimal concentration was therefore 3.0 mmol L^−1^.

#### 2.3.4. Effect of Type and Volume of Elution Solvent

**Figure 7:**
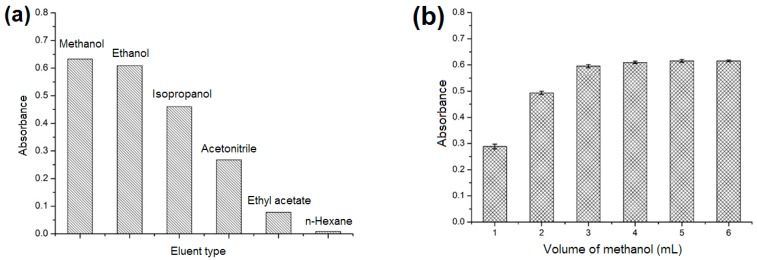
Optimization of (**a**) elution solvent; (**b**) elution volume. Analyte concentration: 3.0 mmol L^−1^; sample volume: 2.0 mL.

The nature of the elution solvent plays an important role in the effective desorption of analyte from adsorbent. The influence of elution solvent on desorption was evaluated under the optimum conditions using six common organic solvents: Methanol, Ethanol, Isopropanol, Acetonitrile, Ethyl acetate, and *n*-Hexane. As shown in Figure 7a, the maximum absorbance of the eluate was obtained when using methanol as the desorption solvent. Therefore, methanol was adopted as the optimal desorption solvent. The volume of methanol was also optimized because the sorption analyte cannot be completely eluted by a small amount of methanol, and long volatilization time will be taken when excessive methanol is used. As presented in Figure 7b, the absorbance of the eluate increased as the volume of methanol was increased from 1 to 4 mL, and no significant changes were observed as the volume was increased further. Consequently, 4 mL of methanol was sufficient for the analytes to be completely eluted and was chosen as the optimized volume.

### 2.4. Reproducibility and Reusability of the SPE Column

**Figure 8:**
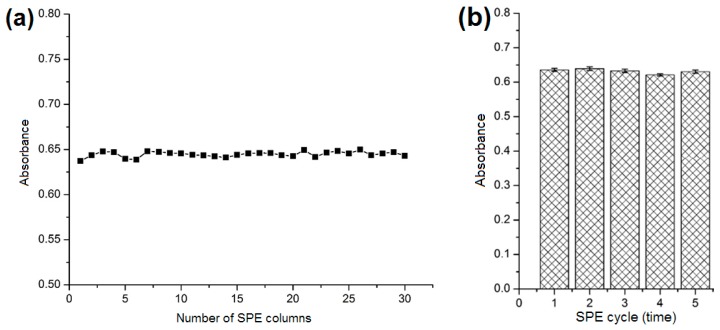
(**a**) Reproducibility and (**b**) reusability of the SPE column.

To evaluate the reproducibility of the SPE column, the sample solution (3 mmol L^−1^, 2 mL) was added to each of 30 SPE cartridges filled with different batches of [Co(l-tyr)]*_n_*(l-tyrCo) particles. As shown in Figure 8a, the differences in the absorbance values of the eluates among the 30 columns were very small, indicating the good reproducibility of the SPE column. The reusability of the SPE column was also investigated. After each SPE experiment, the column was washed sequentially with methanol (10 mL) and *n*-hexane/isopropanol (70/30, *v*/*v*, 10 mL), and then reused for the next extraction cycle. As presented in Figure 8b, no obvious change in absorbance of the eluate was observed after five SPE cycles, indicating good reusability of the SPE column.

### 2.5. Method Validation

**Figure 9:**
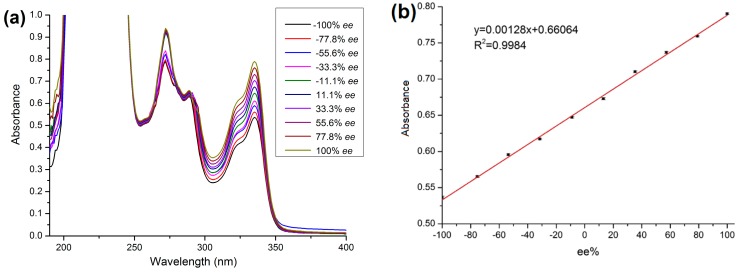
(**a**) UV-visible spectra of the eluates after extraction of (±)-1,1′-bi-2-naphthol at various *ee* values; (**b**) standard curve showing the linearity of *ee%* with the absorbance of the eluates.

Solutions of (±)-1,1′-bi-2-naphthol with varying *ee* values (from −100% to +100%) in *n*-hexane/isopropanol (70:30, *v*/*v*, 3 mmol L^−1^) were prepared by mixing solutions of (*R*)-1,1′-bi-2-naphthol and (*S*)-1,1′-bi-2-naphthol. The solutions (2.0 mL) were passed through the SPE columns under the optimum conditions. The absorbance values of the eluates were determined by UV-visible spectrophotometry (Figure 9a), and the absorbance data at 335 nm were collected. All of the data were fitted by computer and all possible functions tried. An excellent linear relationship between *ee* and the absorbance of the eluate (*R*^2^ = 0.9984) was obtained at the concentration of 3 mmol L^−1^ (Figure 9b), which enabled the calculation of *ee* values for different (±)-1,1′-bi-2-naphthol samples. The selectivity of the MOF for each enantiomer was studied. The adsorption of (*S*)-1,1′-bi-2-naphthol by the MOF was greater than that of (*R*)-1,1′-bi-2-naphthol. The absolute recoveries were 62% for (*R*)-1,1′-bi-2-naphthol and 98% for (*S*)-1,1′-bi-2-naphthol under the developed conditions.

Given that an enantiomerically pure of l-tyrosine was used as ligand in the synthesis, the synthesized [Co(l-tyr)]*_n_*(l-tyrCo) is chiral. The 2D structure of [Co(l-tyr)]*_n_*(l-tyrCo) showing cobalt oxide layers pillared with L-tyrosine linkers and produced large chiral pores (Figure 1a). The influence of the chiral microenvironment on the enantioselective adsorption is complicated. It is difficult to completely understand the mechanism of interaction between the MOF and the two enantiomers. The reason for the selective adsorption of (*S*)-1,1′-bi-2-naphthol may be the high degree of space matching between (*S*)-1,1′-bi-2-naphthol and the chiral structures of the crystal resulting in different forces.

To verify the accuracy of the methodology, solutions of (±)-1,1′-bi-2-naphthol at a variety of *ee* values were determined by this method. The absorbances of the eluates were determined and the *ee* values were calculated using the standard curve (Table 1). Compared with the actual *ee* values measured by chiral HPLC, this method had an average error of 2.26%. This method can therefore be used for evaluation of the *ee* of (±)-1,1′-bi-2-naphthol with good accuracy.

## 3. Materials and Methods

### 3.1. Reagents and Materials

All chemicals and reagents used in this study were at least of analytical grade. (*R*)-1,1′-Bi-2-naphthol and (*S*)-1,1′-bi-2-naphthol were purchased from Sigma-Aldrich (St. Louis, MO, USA). Co(CH_3_COO)_2_∙4H_2_O, l-tyrosine, and NaOH were purchased from Adamas-beta (Shanghai, China), and were used for the synthesis of MOF, [Co(l-tyr)]*_n_*(l-tyrCo). HPLC grade methanol, ethanol, isopropanol, acetonitrile, ethyl acetate, and *n*-hexane were obtained from TEDIA (Fairfield, OH, USA). Ultrapure water (18.2 Ω cm) was produced with an ELGA LabWater water purification system (High Wycombe, UK). The empty SPE cartridges (0.2000 g, 3 mL, polypropylene) with frits (20 μm porosity) were purchased from SEPAX Technologies Inc. (Suzhou, China).

### 3.2. Instrumentations

Powder X-ray diffraction (PXRD) patterns were recorded on a D/max-3B diffractometer (Tokyo, Japan) using Cu K_α_ radiation. Scanning electron microscopy (SEM) images were recorded on a FEI Quanta FEG 650 scanning electron microscope (Hillsboro, OR, USA). The absorbance of eluate was determined on a TU-1901 UV/Vis spectrophotometer (Beijing Purkinje General Instrument Co., Ltd., Beijing, China). Commercial ChiralPak OD-H (250 mm × 4.6 mm, Daicel, Shanghai, China) and a Shimadzu HPLC system (Tokyo, Japan) consisting of an LC-15C HPLC pump and SPD-15C UV/Vis detector were used for the actual *ee* analyses.

### 3.3. Synthesis of [Co(l-tyr)]_n_(l-tyrCo)

[Co(l-tyr)]*_n_*(l-tyrCo) was synthesized according to the method of Rocha et al. [40]. Typically, Co(CH_3_COO)_2_∙4H_2_O and l-tyrosine in a molar ratio of 1:2 were mixed with ultrapure water (20 mL) in a Teflon-lined bomb and stirred with a magnetic bar. The pH of the mixture was adjusted to 9~10 by adding aqueous solution of NaOH (1 mol L^−1^). The bomb was sealed and heated at 130 °C for 3 days. After cooling to room temperature, the mixture was filtered and the purple crystals were washed thoroughly with distilled water and ethanol. Finally, the solid was dried at 100 °C. 

### 3.4. Preparation of SPE Columns

The synthesized crystals of [Co(l-tyr)]*_n_*(l-tyrCo) were large and heterogeneous, which made them unsuitable for direct use as adsorbent in the preparation of SPE columns. The crystals were therefore milled and a suitable particle size was selected by preparing an ethanol suspension. Subsequently, each SPE cartridge was packed with the selected crystal particles (0.2000 g) using polypropylene frits at each end of the cartridge to keep the packing in place. The outlet tip of the cartridge was connected to a vacuum pump, and methanol was continuously added at the inlet end. 

### 3.5. SPE Procedures

Before the extraction experiment, the crystals of the [Co(l-tyr)]*_n_*(l-tyrCo) packed cartridge were preconditioned by washing with methanol (5 mL) and activated with sample solvent (5 mL). A known volume of the sample was passed through the activated cartridge by gravity action. To remove residual sample on the surface of the crystals, additional sample solvent (5 mL) was added after the sample solution had completely passed through. Subsequently, the cartridge was eluted with methanol (4 mL). The eluate was volatilized, diluted with methanol to a final volume of 4 Ml, and the absorbance was determined by UV-visible spectrophotometry.

### 3.6. Calculation of ee Value

The *ee* value of the sample was calculated using the following formula:

$$ee\%=\frac{S-R}{S+R}\;\times \;100\%\;$$

(*R*, *S*: Concentration/peak area, respectively).

Logically, *ee* values of +100% and −100% represent enantiomerically pure of (*S*)-1,1′-bi-2-naphthol and (*R*)-1,1′-bi-2-naphthol, respectively, and an *ee* value of 0% indicates racemate.

## 4. Conclusions

In this work, we reported a novel and simple method for the determination of *ee* value by SPE using a chiral MOF as sorbent. After adsorption and desorption, a standard curve was established that demonstrated a good linear relationship between *ee* and absorbance of the eluate. The *ee* value of (±)-1,1′-bi-2-naphthol can be rapidly calculated after SPE using the standard curve.

## Figures and Tables

**Table 1:** Enantiomeric excess values of (±)-1,1′-bi-2-naphthol calculated from the SPE-based assay.

Sample	Absorbance	Experimental *ee* (%)	Actual *ee* (%)	Absolute Error (%)	Average Error (%)
1	0.5566	−81.25	−84.41	3.16	2.26
2	0.6022	−45.67	−43.34	2.33	
3	0.6582	−1.93	−0.87	1.06	
4	0.7098	38.41	41.79	3.38	
5	0.7621	79.24	77.89	1.35	
